# Purpuric rash after starting hemodialysis—not the immediate suspect: a case report and literature review

**DOI:** 10.3389/fneph.2025.1593915

**Published:** 2025-06-23

**Authors:** George Jiries, Olga Vdovich, Ashraf Badran, Etty Kruzel-Davila

**Affiliations:** ^1^ Nephrology Department, Galilee Medical Center, Nahariya, Israel; ^2^ Azrieli Faculty of Medicine, Bar-Ilan University, Zefat, Israel

**Keywords:** hemodialysis, scurvy, vitamin C deficiency, end-stage kidney disease (ESKD) purpuric rash, case report

## Abstract

**Background:**

Vitamin C deficiency is an underrecognized yet prevalent concern in hemodialysis patients, driven by dietary restrictions, increased oxidative stress, and vitamin losses during dialysis. While supplementation could mitigate deficiency-related complications and reduce inflammation and oxidative damage, clinical implementation remains limited due to concerns about oxalosis and potential pro-oxidative effects.

**Case presentation:**

We report the case of a 74-year-old female with End-Stage Kidney Disease (ESKD) secondary to diabetic nephropathy who developed scurvy after prolonged hemodialysis. She presented with unintended weight loss, gingival bleeding, and recurrent pulmonary edema. Physical examination revealed characteristic dermatological findings, including perifollicular erythema predominantly on the lower extremities. Laboratory testing confirmed severe vitamin C deficiency, with serum levels below the detection limit of 4 mg/L, along with hypoalbuminemia and elevated inflammatory markers. Nutritional assessment indicated adherence to standard hemodialysis dietary restrictions, likely exacerbating deficiency.

**Intervention and outcomes:**

Oral vitamin C supplementation resulted in significant clinical improvement, including resolution of dermatological manifestations, cessation of gingival bleeding, improvement in cardiac function, and without recurrence of pulmonary edema episodes, with no adverse effects observed.

**Conclusion:**

This case highlights the importance of considering scurvy in hemodialysis patients, particularly those with inflammation and restrictive dietary patterns. It underscores the clinical manifestations of vitamin C deficiency, its potential cardiovascular implications, and the need to revisit supplementation guidelines in this population. The findings support the safe and effective use of vitamin C supplementation in reversing deficiency-related complications while emphasizing the broader consideration of routine vitamin C supplementation in hemodialysis patients, even in the absence of overt clinical manifestations.

## Introduction

Vitamin C deficiency is a well-documented concern in patients undergoing hemodialysis, resulting from limited dietary intake, increased oxidative stress, and the removal of water-soluble vitamins during dialysis sessions. Numerous studies and clinical case reports have highlighted both the prevalence and clinical consequences of vitamin C deficiency in this population. While supplementation with vitamin C has the potential to alleviate oxidative stress and modulate inflammatory responses, its safety remains debated due to concerns over the risk about oxalosis and possible pro-oxidative effects.

Current Kidney Disease: Improving Global Outcomes (KDIGO) guidelines recommend considering vitamin C supplementation to achieve the recommended daily intake of at least 90 mg for men and 75 mg for women in patients with chronic kidney disease (CKD) (stages 1-5). However, no stringent guidelines exist for hemodialysis patients due to the ongoing controversy surrounding the balance between potential benefits and adverse effects. Herein, we present a case of scurvy in a hemodialysis patient and critically examine the clinical guidelines and available evidence regarding vitamin C supplementation in this context, with a focus on its risks and therapeutic potential in the hemodialysis setting.

## Case presentation

A 74-year-old female with a history of End-Stage Kidney Disease (ESKD) secondary to diabetic nephropathy had been receiving thrice-weekly maintenance hemodialysis since August, 2020. Over the preceding two years, the patient reported an unintended 8 kg weight loss, likely attributable to decreased appetite and dietary restrictions, including limited intake of fruits and vegetables. Starting in May, 2022, she experienced recurrent episodes of pulmonary edema, necessitating multiple hospital admissions. A transthoracic echocardiogram revealed a reduced left ventricular ejection fraction (LVEF) of 35%, prompting coronary angiography. The angiographic evaluation demonstrated significant stenosis of the left anterior descending (LAD) artery and led to percutaneous coronary intervention with stent placement. Due to recurrent pulmonary edema, the hemodialysis regimen was intensified to four sessions per week. On physical examination, the patient exhibited perifollicular erythema and a purpuric rash localized to the lower extremities, raising clinical suspicion for scurvy. Further anamnesis revealed that the dermatological manifestations had appeared approximately four months after the initiation of hemodialysis and had progressively worsened. Additionally, the patient reported gingival bleeding, a symptom consistent with vitamin C deficiency.

**Figure 1:**
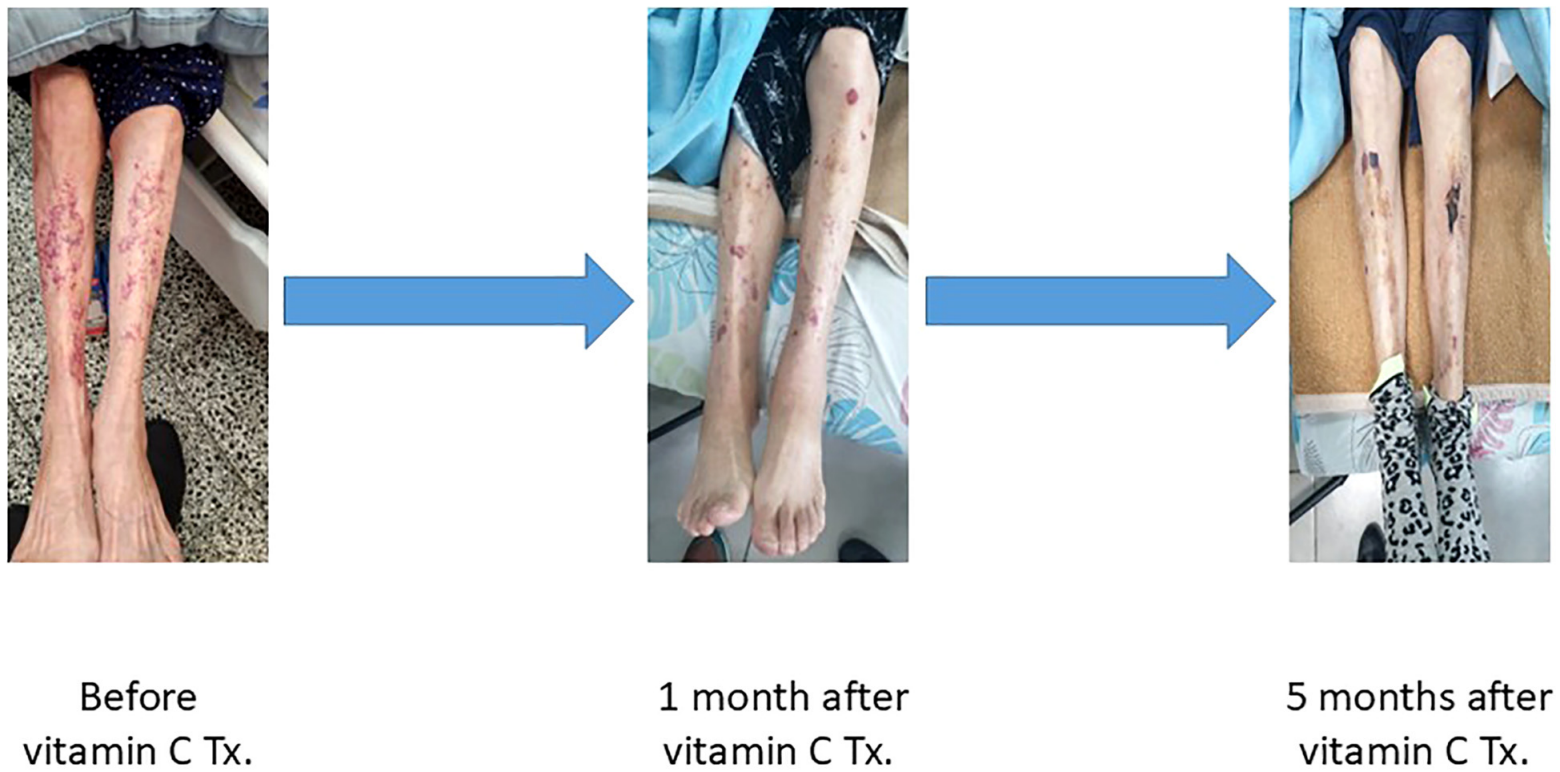
Progressive resolution of scurvy-related skin lesions following vitamin C therapy in a hemodialysis patient. Panels show the lower limbs at three time points: prior to treatment (left), one month after initiating oral vitamin C supplementation (middle), and five months after treatment (right). Marked improvement is evident, with gradual resolution of perifollicular purpura and ecchymoses.

During the current hospitalization, the patient’s vital signs were as follows: blood pressure of 142/85 mmHg, heart rate of 80 beats per minute, respiratory rate of 24 breaths per minute, and oxygen saturation of 92% on room air. She appeared cachectic, with a low body mass index (BMI) of 16.8 kg/m². On physical examination, cardiac auscultation revealed a regular rhythm with normal heart sounds, without any murmurs or pericardial friction rub. Lung examination identified crepitations at the bases, while the abdominal examination was unremarkable. Prominent perifollicular erythema and purpuric rash over both ankles were observed. Prominent perifollicular erythema and a purpuric rash over both ankles were observed. This presentation warranted consideration of a broad differential diagnosis, including vasculitis, calciphylaxis, and thrombocytopenia and/or uremic platelet dysfunction, all recognized complications in patients undergoing hemodialysis. However, the presence of gingival bleeding, unintentional weight loss, the absence of systemic features suggestive of vasculitis, and normal platelet counts guided us to investigate vitamin C deficiency as a potential underlying cause (**Figure 1**).

Laboratory investigations, summarized in **Table 1**, revealed hypoalbuminemia and elevated C-reactive protein (CRP), indicative of an inflammatory state. Given the clinical suspicion of severe vitamin C deficiency, serum vitamin C levels were measured and found to be markedly below the lower detection limit (4 mg/L), as determined by colorimetric assay. In accordance with best practices for hemodialysis patients, the sample was obtained pre-dialysis and midweek, prior to the second dialysis session of the week. This timing was intentionally selected to minimize the effects of dialysis-related vitamin C clearance and to avoid the variability associated with the long interdialytic interval over the weekend, thereby providing a more accurate reflection of the patient’s baseline vitamin C status (1). Based on these findings, a diagnosis of scurvy was established.

**Table 1:** Baseline laboratory parameters upon hospital admission at the time of scurvy diagnosis.

Parameter	Value
Hemoglobin	12 g/dL
White blood cells (WBC)	7.6x10^3^/µL
Platelets (PLT)	308x10^3^/µL
Transferrin Saturation (TSAT)	21%
Ferritin	652 ng/mL
Folic acid	20 nmol/L
Vitamin B12	684 pmol/L
Albumin	3.2 g/dL
Calcium	8.4 mg/dL
Phosphorus	2.3 mg/dL
Parathyroid Hormone (PTH)	339 pg/mL
C Reactive Protein (CRP)	220.1mg/l
Cholesterol, total	193 mg/dl
Triglycerides	135 mg/dl
HDL-cholesterol	50.1 mg/dl
LDL cholesterol	115.9 mg/dl
Non HDL cholesterol	143
pH	7.2
Bicarbonate	20 nmol/L
Alkaline phosphatase	99U/L
Vitamin C plasma	<4 mg/L

Oral vitamin C therapy was initiated at a dose of 500 mg daily for one month. After four weeks of treatment, there was a significant improvement in the perifollicular purpuric rash and resolution of gingival bleeding. (**Figure 1**) Following 1 month of high-dose vitamin C therapy, the dose was reduced to 250 mg every other day due to concerns regarding the risk of oxalosis, with continued clinical improvement. Additionally, a follow-up echocardiogram performed five months after initiating therapy demonstrated improved LVEF from 35% to 45%. Importantly, no further hospitalizations for pulmonary edema occurred following the initiation of vitamin C therapy. The reduced dose of 250 mg every other day was maintained, supporting sustained clinical recovery.

## Discussion

### Vitamin C deficiency: evolution, function, and clinical impact

Humans lack a functional gene encoding gluconolactone oxidase, the enzyme responsible for endogenous vitamin C synthesis. This loss is believed to have occurred approximately 45 million years ago, possibly as an adaptive evolutionary response. One hypothesis suggests that inactivating this enzyme reduced hydrogen peroxide production, mitigating oxidative stress. Another proposes that reduced ascorbic acid levels may have enhanced activation of hypoxia-inducible factor (HIF), offering a selective advantage under low-oxygen conditions (2–4). As a result, vitamin C must be obtained through dietary intake, primarily from fruits and vegetables (5). Since James Lind’s, 1753 discovery linking citrus intake to scurvy prevention, vitamin C has been recognized as essential for collagen biosynthesis, neurotransmitter production, and immune and endothelial function (5, 6). Ascorbic acid acts as a cofactor in several hydroxylation reactions and contributes to antioxidant defense, although its unique role in oxidative protection remains debated due to redundancy in other antioxidant pathways (2, 5).

Clinical manifestations of scurvy include hemorrhagic signs, hyperkeratosis of hair follicles, generalized weakness (often described as hypochondriasis), and anemia (5). Some of these symptoms are non-specific and may overlap with those associated with CKD (7). Hence, scurvy is often underdiagnosed in this population.

### Critical appraisal of vitamin C supplementation in hemodialysis

Patients undergoing hemodialysis frequently experience vitamin C deficiency due to several contributing factors, including dietary restrictions, the loss of water-soluble vitamins during hemodialysis, and chronic oxidative stress that depletes antioxidant reserves such as vitamin C. Notably, hemodiafiltration (HDF) enhances the removal of vitamin C compared to conventional hemodialysis, owing to its higher convective clearance (8). As a result, low plasma concentrations of vitamin C are more common in hemodialysis patients than in the general population and are often associated with poor nutritional status (1, 9). The KDIGO guidelines recommend considering vitamin C supplementation in patients with CKD stages 1-5 to meet the daily requirements of 90 mg for men and 75 mg for women (10). However, the use of vitamin C supplementation in hemodialysis patients remains a subject of debate (11). While supplementation may effectively address vitamin C deficiency and mitigate oxidative stress, potentially improving patient outcomes, concerns persist regarding the risk of adverse effects, including oxalosis, detrimental cardiovascular outcomes, and heightened inflammatory responses. This tension between potential benefit and harm underscores the ongoing debate. In this review, we critically evaluate these competing considerations in hemodialysis management.

### Vitamin C, iron bioavailability, and anemia in hemodialysis patients

Vitamin C enhances the absorption of non-heme iron by reducing the insoluble ferric form (Fe³^+^) to the more bioavailable ferrous form (Fe²^+^). Additionally, it facilitates the mobilization of stored iron from ferritin and hemosiderin, thereby improving overall iron availability (7). Multiple studies have demonstrated that vitamin C, by enhancing non-heme iron absorption and promoting the mobilization of stored iron, significantly improves the efficacy of erythropoiesis-stimulating agents (ESAs) in the treatment of anemia among hemodialysis patients. Oral vitamin C therapy has also been associated with a statistically significant reduction in both hepcidin and high-sensitivity C-reactive protein (hs-CRP) levels after three months (12). These effects supports more efficient erythropoiesis, contributing to improved management of anemia in this population (13–16). However, the sample sizes in these studies were insufficient to adequately assess the safety of this intervention. Consequently, the KDIGO guidelines do not endorse the use of vitamin C as an adjunct to ESA therapy in hemodialysis patients (17).

### The dual role of Vitamin C in oxidative stress and inflammation in hemodialysis

Vitamin C is a potent antioxidant, capable of neutralizing free radicals, thereby reducing oxidative stress and preventing cellular damage. It plays a protective role by scavenging reactive oxygen species (ROS) and mitigating lipid peroxidation, which contributes to its anti-inflammatory properties (9). Hemodialysis patients are subjected to heightened oxidative stress due to the underlying CKD and the dialysis process (7). This persistent inflammatory state increases the risk of cardiovascular complications and susceptibility to infections (18). By scavenging ROS, vitamin C may reduce oxidative damage to cells, including endothelial cells, and help restore antioxidant balance. In a cohort study involving hemodialysis and peritoneal dialysis patients, vitamin C deficiency was associated with elevated inflammatory markers, suggesting a link between inadequate vitamin C levels and heightened inflammation (19). Yang et al. reported that intravenous vitamin C administration mitigated hemodialysis-induced oxidative stress, evidenced by a reduction in hemolysis, lipid peroxidation, and pro-inflammatory cytokines (20). In addition, vitamin C supplementation was associated with an increase in paraoxonase (PON1) activity and a reduction in advanced glycation end products (AGEs) and lipid hydroperoxide levels (21). Other studies have demonstrated that both oral and intravenous vitamin C supplementation can reduce inflammatory markers in hemodialysis patients (22–24). However, one study found that short-term oral vitamin C supplementation did not significantly affect oxidative stress or inflammation markers in hemodialysis patients (25).

Despite these potential benefits, although vitamin C is primarily known for its antioxidant properties, at high concentrations, it can exhibit pro-oxidant behavior, leading to the production of reactive ROS and heightened inflammation. This paradoxical effect could potentially exacerbate oxidative stress in hemodialysis patients, thereby negating the anticipated benefits of supplementation. De Vriese et al. reported that oral vitamin C supplementation in hemodialysis patients resulted in increased lipid peroxidation, as indicated by elevated malondialdehyde levels, particularly in those with high serum ferritin concentrations (26). In addition, vitamin C facilitates the reduction of ferric iron (Fe³^+^) to ferrous iron (Fe²^+^), which may contribute to pro-oxidant hydroxyl radical formation through Fenton’s reaction, a key contributor to the generation of ROS (9). Supporting this theory, Chen et al. demonstrated that vitamin C supplementation enhanced free radical production in patients with elevated ferritin concentrations (27). Other studies have also highlighted ascorbic acid’s potential role as a pro-oxidant and pro-inflammatory agent when administered intravenously in conjunction with ferric iron, underscoring the risks associated with combined therapy in enhancing oxidative stress (28). Thus, while restoring vitamin C levels may enhance erythropoiesis and antioxidant function, these benefits may be offset by the risk of oxidative tissue injury. These findings underscore the importance of carefully evaluating the risks and benefits of vitamin C supplementation in hemodialysis patients (26).

### Oxalosis risk with vitamin C supplementation in hemodialysis

One of the primary concerns regarding vitamin C supplementation in hemodialysis patients is the risk of oxalosis, as vitamin C is metabolized to oxalate, a byproduct that cannot be effectively cleared in individuals with impaired renal function. Hemodialysis or HDF partially removes oxalate from the bloodstream (29). This limited clearance is a significant concern for patients receiving vitamin C supplementation, as the accumulation of oxalate can lead to complications such as oxalosis and associated tissue damage. Oxalate accumulation can lead to systemic oxalosis, potentially resulting in serious complications such as nephrocalcinosis, vascular calcifications, and damage to various organ systems (2). Furthermore, elevated serum oxalate levels have been linked to an increased risk of cardiovascular events and sudden cardiac death in patients undergoing dialysis (30). This association underscores the importance of monitoring and managing oxalate levels in this population, particularly in the context of vitamin C supplementation and its contribution to oxalate accumulation. Intravenous vitamin C administered in moderate doses of 250–500 mg per week has been associated with increased in plasma oxalate levels, which may exceed the supersaturation threshold for calcium oxalate in some patients (31). Therefore, it is essential to monitor oxalate levels in patients receiving vitamin C supplementation to mitigate potential risks associated with oxalate accumulation and its adverse effects (29, 30, 32, 33).

### Vitamin C and cardiovascular risk in dialysis patients

Cardiovascular disease remains a leading cause of morbidity and mortality among dialysis patients (34). Vitamin C may enhance endothelial function and reduce arterial stiffness, potentially lowering the risk of cardiovascular events this population (9). Two prospective studies have reported that low plasma levels of vitamin C are predictive of adverse cardiovascular outcomes in maintenance hemodialysis patients. However, as with all association studies, causality cannot be established (35, 36).

In prospective studies, vitamin C supplementation has not demonstrated a significant impact on quality of life (QoL), mortality, or hospitalizations in hemodialysis patients (37, 38). Additionally, vitamin C is metabolized to oxalate, which cannot be effectively cleared in patients with impaired renal function. A *post hoc* analysis of the randomized German Diabetes Dialysis (4D) Study identified that elevated serum oxalate levels as a novel risk factor for cardiovascular events and sudden cardiac death in dialysis patients (30). Consequently, vitamin C supplementation may elevate the risk of cardiovascular events. Accordingly, current evidence is insufficient to support firm recommendations for the use of vitamin C to improve cardiovascular outcomes in this population (10).

### Vitamin C and DNA integrity in hemodialysis patients

Theoretically, vitamin C protects against DNA mutations by neutralizing ROS that can damage cellular components, including DNA. However, there is currently no conclusive evidence demonstrating that vitamin C has a protective effect of in terms of strand breaks, micronuclei formation, or chromosomal aberrations (3). In hemodialysis patients, as they are prone to elevated oxidative stress, vitamin C may help prevent oxidative damage to lymphocytes and other immune cells, thereby supporting immune function and potentially reducing the risk of malignancies (9). Tarng et al. demonstrated that vitamin C supplementation in chronic hemodialysis patients significantly reduced lymphocyte levels of 8-hydroxy-2’-deoxyguanosine (8-OHdG), a biomarker of oxidative DNA damage and intracellular ROS production. Furthermore, the study found that vitamin C supplementation upregulated the expression of the 8-oxo guanine-DNA glycosylase 1 (hOGG1) gene, which encodes a DNA glycosylase critical for the base excision repair pathway targeting 8-OHdG in oxidatively damaged DNA (39). Thus, ascorbic acid may protect lymphocyte DNA either by inhibiting the formation of ROS or scavenging these harmful molecules. Nevertheless, current evidence supporting a reduction in cancer incidence associated with vitamin C supplementation in hemodialysis patients remains insufficient (10).

### Preventing scurvy in hemodialysis patients

Hemodialysis patients are at an elevated risk of developing scurvy due to several contributing factors, including restricted dietary intake of fruits and vegetables, the loss of vitamin C during the dialysis process, and increased oxidative stress. To prevent scurvy in this population, it is essential to implement strategies that maintain adequate vitamin C levels. These strategies may involve dietary modifications, appropriate supplementation, and regular monitoring of vitamin C status, particularly when clinical suspicion of scurvy arises (7, 10, 36, 40). Supplementation can effectively prevent or treat scurvy, as demonstrated in our patient (7, 10).

### Uncertainty and guidelines for vitamin C supplementation in hemodialysis

Despite the theoretical benefits of vitamin C supplementation, robust evidence regarding its long-term effects, safety, and efficacy in hemodialysis patients remains limited. This uncertainty has led to cautious recommendations from nephrology guidelines, and a lack of consensus on the optimal dosing and duration of supplementation (10). The KDIGO guidelines recommend considering vitamin C supplementation for CKD patients, including those on dialysis, to achieve the recommended daily intake. However, these guidelines do not endorse high-dose supplementation due to associated risks, particularly the potential for oxalosis. Current recommendations support moderate dosing (≤ 100 mg/day) in hemodialysis patients to minimize the risk of excessive vitamin C accumulation and related complications. Further clinical studies are needed to establish definitive guidelines for the optimal dosage and duration of supplementation, particularly concerning cardiovascular outcomes and oxidative stress management.

## Conclusion

We present a case of a hemodialysis patient diagnosed with scurvy, a severe manifestation of vitamin C deficiency. Despite the patient’s dietary restrictions and heightened oxidative stress, vitamin C supplementation effectively corrected the deficiency and resulted in marked clinical improvement, including resolution of the purpuric rash and gingival bleeding. Furthermore, we propose that the observed improvement in cardiac function and the reduction in hospitalizations due to pulmonary edema may have been influenced, at least in part, by vitamin C supplementation. This hypothesis is supported by a recent meta-analysis demonstrating that vitamin C administration significantly increased LVEF in both cardiac and non-cardiac patients, with the greatest benefit observed in those with lower baseline LVEF (41). However, despite the temporal association, caution is warranted in drawing causal conclusions, as cardiac outcomes in dialysis patients are influenced by multiple factors, including coronary angioplasty, increased dialysis frequency, medication adjustments, and the possibility of spontaneous recovery.

This case highlights that scurvy is often underdiagnosed in hemodialysis patients due to the overlapping symptoms of scurvy and uremia, which can obscure clinical recognition and contribute to significant cardiovascular complications. Therefore, maintaining a low threshold for clinical suspicion is essential to ensure timely diagnosis and treatment of this preventable and potentially devastating condition.

This case not only illustrates the clinical consequences of overt vitamin C deficiency but also underscores the broader clinical challenge of managing vitamin C status in hemodialysis patients. Vitamin C supplementation in this population presents a complex interplay of benefits and risks. While it may help reduce oxidative stress and inflammation, as potentially reflected in the patient’s improved cardiac function, excessive supplementation can lead to adverse outcomes such as oxalosis and pro-oxidant effects. Given the lack of conclusive evidence, clinicians should carefully assess the need for supplementation, especially in patients presenting with suggestive symptoms. Moderate dosing, coupled with regular monitoring for oxalate accumulation and adverse effects, is advisable. Patients with malabsorption or chronic inflammatory states may be at increased risk of deficiency and should be evaluated accordingly. Further research is essential to guide evidence-based use of vitamin C in this vulnerable population and to prevent both overt deficiency and potential harm from overcorrection.

### Study limitation

We acknowledge that metaphosphoric acid, which prevents the spontaneous oxidation and degradation of ascorbic acid, was not used in the colorimetric assay. This omission may have led to an underestimation of the patient’s true serum vitamin C level, potentially affecting the accuracy of the laboratory result. Additionally, due to logistical challenges, repeat measurements were not conducted to monitor vitamin C and oxalate levels over time. Despite this methodological limitation, the prompt and marked clinical improvement observed following vitamin C supplementation, specifically the resolution of hallmark symptoms such as gingival bleeding and purpura, provides strong support for the diagnosis of scurvy and the biological relevance of the intervention. Oxalate levels were not monitored, as the patient received a moderate dose of vitamin C for a limited duration. However, the potential risk of oxalate accumulation associated with supplementation remains an important consideration, particularly in cases requiring prolonged or high-dose therapy, as elaborated in the Discussion section.

### Patient perspective

Clinical improvement was observed within several weeks of initiating vitamin C supplementation, with complete resolution of gingival bleeding and gradual fading of the purpuric rash and bruising on the lower extremities. Notably, the patient experienced no further episodes of shortness of breath requiring hospitalization, an issue that had been recurrent prior to treatment. This improvement was sustained five months later, with the patient continuing to report significant symptom relief and a marked enhancement in quality of life, highlighting the lasting impact of this simple yet effective nutritional intervention.

## Data Availability

The data supporting the conclusions of this article are available from the corresponding author upon reasonable request. Due to patient confidentiality and institutional restrictions, data are not publicly available.
